# A Rare Case of Lyme Neuroborreliosis Presenting as Bannwarth Syndrome in Texas

**DOI:** 10.7759/cureus.109422

**Published:** 2026-05-22

**Authors:** Aabhishkar Bhattarai, Manmeet Kaur, Vijaya Valaparla, Xiangping Li

**Affiliations:** 1 Department of Neurology, University of Texas Medical Branch, Galveston, USA

**Keywords:** bannwarth syndrome, bilateral facial palsy, lower extremity radiculopathy, lyme neuroborreliosis, lymes

## Abstract

Lyme disease is a spirochete infection acquired after inoculation of *Borrelia burgdorferi *with the bite of an Ixodes tick, commonly encountered in the Northeastern United States, usually characterized by erythema migrans, arthritis, and meningitis. Bannwarth syndrome is an uncommon manifestation of Lyme neuroborreliosis (LNB) in the United States. It can present with various clinical features, which often make the diagnostic process challenging. In our case, the subacute onset of flaccid leg weakness with diminished deep tendon reflexes and facial weakness mimicked Guillain-Barré syndrome (GBS). Physicians need to be aware of the rare neurological manifestations of Bannwarth syndrome, especially in non-endemic areas such as Texas. Here we present a rare case of Lyme neuroborreliosis (LNB) manifesting as a painful meningoradiculitis (Bannwarth syndrome) with radicular pain, paresis, and cranial nerve involvement in Texas.

## Introduction

Lyme disease is a tick-borne spirochetal infection caused by *Borrelia burgdorferi *and related *Borrelia *species. It is the most commonly reported vector-borne illness in the United States, with recent estimates suggesting that approximately 476,000 people are diagnosed and treated for Lyme disease annually. However, its geographic distribution remains highly uneven, with the majority of cases occurring in the northeastern, mid-Atlantic, and north-central United States. Texas is considered a low-incidence state for Lyme disease; from 2000 to 2020, the Texas Department of State Health Services reported an average of 85 human cases per year, and more recent state surveillance reported 27 cases in 2023, 21 cases in 2024, and 19 provisional cases in 2025 [1,2].

Lyme neuroborreliosis refers to involvement of the central or peripheral nervous system by Lyme borreliosis. Neurologic manifestations may be classified temporally as early or late disease. Early disseminated Lyme neuroborreliosis typically occurs within weeks to months of infection and may present with lymphocytic meningitis, cranial neuritis, radiculoneuritis, or painful meningoradiculitis. Late neuroborreliosis is less common and may present more indolently with central or peripheral nervous system involvement. Bannwarth syndrome is a form of early Lyme neuroborreliosis classically defined by painful lymphocytic meningoradiculitis, often accompanied by radicular pain, motor weakness, sensory symptoms, and cranial neuropathy. 

This case adds clinical value beyond its occurrence in a low-incidence geographic region because the patient’s presentation closely mimicked Guillain-Barré syndrome (GBS), with subacute flaccid lower extremity weakness, diminished deep tendon reflexes, radicular pain, and facial nerve involvement. Such overlap can delay diagnosis, particularly in non-endemic areas where Lyme neuroborreliosis may not be initially considered. By highlighting Bannwarth syndrome as a treatable mimic of inflammatory polyradiculoneuropathy, this case emphasizes the importance of maintaining Lyme neuroborreliosis in the differential diagnosis of painful radiculopathy, cranial neuropathy, and acute or subacute weakness, even in regions such as Texas where reported Lyme disease incidence is low.

## Case presentation

A 73-year-old Caucasian male, right-handed, with a past medical history of hypertension (HTN), prediabetes, asthma, bilateral knee osteoarthritis, and noise-induced sensorineural hearing loss (left>right), presented to the hospital with chief complaints of back pain, generalized weakness, and insomnia for 2 weeks, left leg weakness for 5 days, along with facial weakness for 2 days. The patient complained of diffuse back pain from the mid-thoracic to the lumbar area for 2 weeks. In addition to the low back pain with radiation to the left buttock and knee, he complained of thoracic pain radiating to the left subscapular region. 

He saw his primary care provider for acute back pain and was diagnosed with sciatica. Despite multiple medications for back pain, including meloxicam, acetaminophen, gabapentin, and cyclobenzaprine, his pain often intensified through the night, and he suffered insomnia and generalized malaise.

He denied having neurological symptoms other than a feeling of numbness in his feet 4-6 months ago. He also denied having any recent fever, chills, night sweats, skin rash, headache, vision changes, upper respiratory infection, or diarrhea.

On examination, vital signs were stable, and the patient was afebrile. Neurological examination revealed intact mentation, asymmetrical bilateral lower motor neuron facial nerve weakness (right>left) with House-Brackmann score of 3, bilateral sensorineural hearing loss (chronic), weakness in hip flexors, knee flexors, and extensors of the left lower extremity (4/5) and normal strength in all muscle groups in right leg, bilateral reduced deep tendon reflexes in the patella, and absent Achilles reflex. Sensory examination was remarkable for a patchy loss of sensation below the left nipple in an oval pattern, as well as reduced pinprick and temperature sensation in a stocking distribution up to the lower 1/3rd of the shin. Proprioception was intact. No skin rash or meningeal signs were noted. Routine labs were unremarkable, including complete blood counts, basic metabolic profile, magnesium, calcium, urinalysis, and urine drug screen.

Initial workup with CT head and CT angiogram was unremarkable. Peripheral inflammatory markers like erythrocytic sedimentation rate and C-reactive protein were within normal limits. Cerebrospinal fluid analysis (Table 1) revealed protein 215 mg/dL, glucose 52 mg/dL, WBC 226 cells/mm³ with lymphocytic predominance, and RBC 4,000 cells/mm³, suggestive of a traumatic tap. Using a correction factor of 1 WBC per 500-1,000 RBCs, the corrected CSF WBC count was approximately 218-222 cells/mm³; therefore, the degree of pleocytosis remained clinically significant and could not be explained by traumatic contamination alone. Cerebrospinal fluid was negative for PCR for meningitis encephalitis panel, which included *E. coli*, *Streptococcus agalactiae*, *Streptococcus pneumoniae*, *Listeria monocytogenes*, *Neisseria meningitides*, cytomegalovirus, enterovirus, herpes simplex virus 1 and 2, human herpesvirus 6, human parechovirus, varicella-zoster virus, and *Cryptococcus neoformans*/*gattii*.

**Table 1:** Cerebrospinal Fluid (CSF) analysis findings

Parameter	Value	Reference Range
Appearance	Slightly bloody	Clear
WBC count, cells/µL	226	0-5
RBC count, cells/µL	4000	<10
% Polymorphonuclear cells	3	<6
% Lymphocytes	80	40-80
% Reactive Lymphocytes	9	
% Macrophages	74	15-45
% Plasma cells	3	
Borrelia burgdorferi Antibodies, IgM & IgG by enzyme-linked immunosorbent assay (ELISA)	IgG 3.75 IV; IgM 4.26 IV	Ref interval: <=0.90
Borrelia burgdorferi VlsE1/pepC10 Antibodies, Total by ELISA With Reflex to IgM and IgG by ELISA (Modified Two-Tier Testing)	4.80 IV	Ref interval: <=0.90
Lyme Modified 2-Tier Testing Interpretation	Positive	Negative
Autoimmune reflex panel	Negative	Negative
Paraneoplastic panel	Negative	Negative
West Nile virus IgG by ELISA	0.70	Ref interval: <=0.90
Lymphocytic Choriomeningitis (LCM) Virus Antibody, IgG	<1:10	Ref interval: <=1.10

Magnetic Resonance Imaging (MRI) (Figure 1) of the brain and spine demonstrated enhancement of the bilateral post-labyrinthine, tympanic, and mastoid segments of the facial nerves. Post-contrast sagittal and axial T1-weighted spinal images showed cauda equina nerve root enhancement, consistent with inflammatory radiculitis. Multilevel degenerative changes were present in the cervical and lumbar spine. A focused nerve conduction study (Figure 2) evaluating F-waves was normal in the bilateral upper and lower extremities.

**Figure 1:**
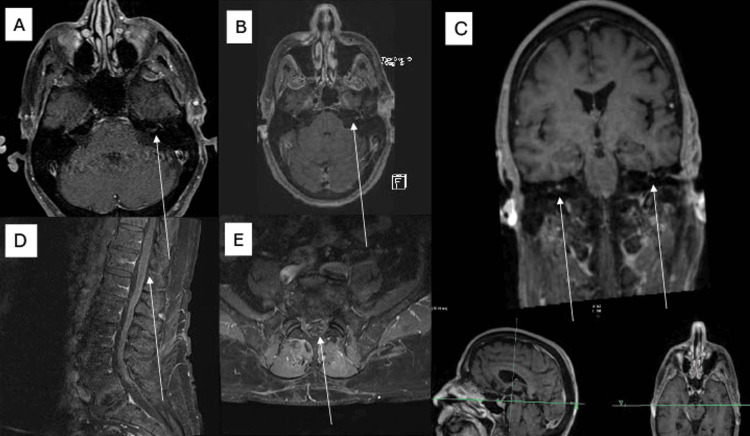
MRI of the brain and spine 1A,1B: Axial T1 postcontrast images demonstrate abnormal but symmetric enhancement of bilateral facial nerves. 1C: Coronal T1 postcontrast images also show abnormal fundibular enhancement of the auricular segment of the bilateral facial nerve. 1D, 1E: Sagittal and axial T1 images, postcontrast, show enhancement of cauda equina nerve roots.

**Figure 2:**
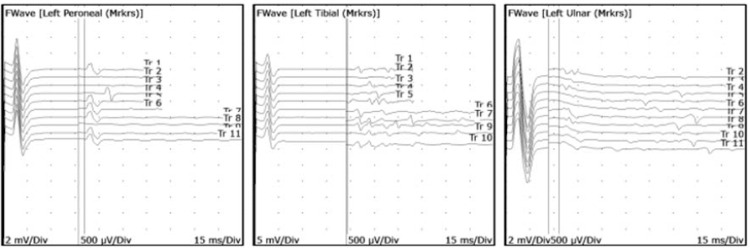
Nerve conduction studies with normal F-wave latencies on left peroneal, left tibial, and left ulnar nerves

On further history exploration, the patient visited Pennsylvania three months before the presentation (in late June). He described moving logs and cutting logs with large chainsaws in the woods. He denied any known tick bites, erythema migrans rash, flu-like symptoms, arthralgia, or other constitutional symptoms.

On day 5 of admission, serum *Borrelia burgdorferi *antibodies (modified two-tier testing) returned positive with an IgM titer of 4.26 (ref interval: <=0.90) and an IgG titer of 3.75 (ref interval: <=0.90).

On day 7, CSF *Borrelia burgdorferi *Antibodies returned positive with a titer of 3.60 (normal <= 0.99) with positive Lyme IgM and IgG. 

The clinically decisive findings supporting Lyme neuroborreliosis presenting as Bannwarth syndrome included severe radicular pain, bifacial palsy, CSF lymphocytic pleocytosis with elevated protein, facial nerve and cauda equina nerve root enhancement, and positive *Borrelia burgdorferi *antibody testing in the CSF with positive Lyme IgM and IgG serology. Alternative etiologies, including West Nile virus and paraneoplastic processes, were excluded with negative testing. Given the patient’s initial presentation with bifacial weakness, paresthesias, limb weakness, and diminished reflexes, an atypical Guillain-Barré syndrome variant, particularly bifacial diplegia with paresthesias, was initially considered. Miller Fisher syndrome was also considered early in the differential diagnosis because multiple cases had recently been identified in the Galveston, Texas area, where this patient was diagnosed; however, the absence of a classic Miller Fisher triad and the patient’s prominent radicular pain made this diagnosis less likely.

Intravenous immunoglobulin (IVIG) was started empirically but discontinued after the CSF profile and subsequent Lyme serologies supported an infectious meningoradiculitis rather than an immune-mediated polyradiculoneuropathy. The clinically decisive findings supporting Lyme neuroborreliosis presenting as Bannwarth syndrome included severe radicular pain, bifacial palsy, CSF lymphocytic pleocytosis with elevated protein, facial nerve and cauda equina nerve root enhancement, and positive *Borrelia burgdorferi *antibody testing in the CSF with positive Lyme IgM and IgG serology. Alternative etiologies, including West Nile virus and paraneoplastic processes, were excluded with negative testing. The patient was treated with oral doxycycline 100 mg twice daily for 28 days, with subsequent clinical improvement.

## Discussion

*Borrelia burgdorferi *(BB) is the most common cause of Lyme disease in the United States. It is transmitted through the bite of Ixodes ticks. Neurological involvement occurs in the early disseminated and late phases of the disease [3]. Lyme disease is most commonly endemic in the Northeastern, mid-Atlantic, and Upper Midwestern United States [4]. Lyme neuroborreliosis can involve the cranial nerves, meninges, peripheral nerves, and nerve roots. Cranial neuritis is a common manifestation, particularly affecting the facial nerve, cranial nerve VII. Patients may also present with meningitis and radicular polyneuritis, leading to Bannwarth syndrome [5]. It typically presents with radicular pain, dermatomal sensory disturbances, and peripheral polyradiculoneuropathy mimicking GBS [6].

Although systemic involvement, including dermatitis, arthritis, and carditis, is often seen, neurological involvement, including radiculitis, is less common in the United States than in European nations [7]. Although facial nerve involvement is most common, other cranial nerves that could be affected are III, IV, VI, and VIII [8]. LNB begins when an Ixodes tick harboring *B. burgdorferi *attaches to a host. Spirochetes disseminate via blood or peripheral nerves, reaching the central nervous system through CSF or meninges, particularly the dura mater. This triggers T cell and glial activation, Interleukin-6 release, oligodendrocyte apoptosis, and neuronal injury. Even after antibiotic treatment, molecular disruptions (e.g., eIF2 pathway changes, non-coding RNA alterations) can persist, and in some cases, spirochete DNA or organisms are found in brain tissue, potentially contributing to post-treatment neurological symptoms [9].

Our patient had several atypical features that contributed to diagnostic delay. He presented in Texas, a low-incidence region for Lyme disease, and travel to an endemic area was identified only after repeated targeted questioning. He denied known tick exposure, erythema migrans, headache, lymphadenopathy, flu-like symptoms, or other early localized Lyme disease features. His bifacial weakness, left lower extremity weakness, paresthesias, and hyporeflexia initially suggested an atypical Guillain-Barré syndrome variant, particularly bifacial diplegia with paresthesias. However, the presence of prominent radicular pain, CSF lymphocytic pleocytosis, and markedly elevated CSF protein supported infectious/inflammatory meningoradiculitis rather than classic GBS, which typically demonstrates albuminocytologic dissociation.

Facial nerve and cauda equina nerve root enhancement further supported cranial neuritis and radiculitis. Lyme neuroborreliosis was diagnosed only after positive Lyme serologies returned, emphasizing that Bannwarth syndrome may be delayed or missed in low-incidence regions when erythema migrans is absent. The patient fulfilled the European Federation of Neurological Societies criteria for definite early Lyme neuroborreliosis: compatible neurologic symptoms with exclusion of alternative causes, CSF pleocytosis, and CSF *Borrelia burgdorferi*-specific antibodies consistent with intrathecal antibody production [10].

Neuroborreliosis is treated with intravenous antibiotics such as ceftriaxone or penicillin G; however, oral doxycycline has demonstrated comparable efficacy to intravenous therapy in selected cases, with adequate central nervous system penetration at a recommended dose of 100 mg twice daily for 2 to 4 weeks [11]. Our patient received a 28-day course of oral doxycycline. At the 2-month follow-up after hospital discharge, he demonstrated interval neurological improvement, including improvement in bifacial weakness (House-Brackmann grade 1) and left lower extremity strength (5/5 in all muscle groups), resolution of left anterior chest numbness, and alleviation of back pain. No progression of weakness, recurrent radicular pain, or new neurological deficits were reported at follow-up [12].

Considering its polymorphic presentation and potential involvement of multiple organ systems, including the skin, nervous system, musculoskeletal system, eyes, and heart, Lyme disease poses a significant diagnostic challenge. Healthcare providers across specialties should recognize Lyme neuroborreliosis as a potential mimic of inflammatory polyradiculoneuropathy, even in non-endemic regions.

## Conclusions

This case highlights the importance of considering Lyme neuroborreliosis in the differential diagnosis of patients presenting with painful radiculopathy, cranial neuropathy, and acute or subacute weakness, even when they present in low-incidence regions such as Texas. In this patient, the infection was likely acquired during prior travel to Pennsylvania, an endemic region, and was later diagnosed after presentation in Texas. This distinction between an imported case and locally acquired infection is clinically important and underscores the need for a careful travel and exposure history. The absence of a known tick bite or erythema migrans should not exclude Lyme neuroborreliosis when the clinical syndrome, CSF profile, neuroimaging findings, and serologic testing are supportive. Early recognition of Bannwarth syndrome as a treatable mimic of atypical Guillain-Barré syndrome variants may prevent diagnostic delay and allow timely antibiotic therapy.
